# Effects of fenfluramine and sigma‐1‐dependent pharmacological and genetic modulation in a mouse kindling model

**DOI:** 10.1002/epi.70270

**Published:** 2026-04-29

**Authors:** Eva‐Lotta von Rüden, Verena Buchecker, Amelie Wagner, Victoria M. Stocker, Liga Zvejniece, Heidrun Potschka

**Affiliations:** ^1^ Institute of Pharmacology, Toxicology, and Pharmacy Ludwig‐Maximilians‐Universität Munich Germany; ^2^ Laboratory of Pharmaceutical Pharmacology Latvian Institute of Organic Synthesis Riga Latvia

**Keywords:** antiseizure medication, E1R, NE‐100, sigma‐1 knockout, temporal lobe epilepsy

## Abstract

**Objective:**

Sigma‐1 is a chaperone protein that serves as a key homeostatic regulator, implicated in neuronal excitability and seizure control. Positive allosteric modulators offer a use‐dependent means to enhance Sigma‐1 activity, potentially with favorable tolerability compared to direct agonists. This study examined the role of sigma‐1 in ictogenesis, seizure spread, and termination, and evaluated whether sigma‐1 targeting could modify progression in the amygdala kindling model.

**Methods:**

Using the mouse amygdala kindling paradigm, we assessed the effects of subchronic administration of the sigma‐1 positive allosteric modulator E1R and of the antiseizure medication fenfluramine on seizure thresholds, severity, duration, and progression of kindling. Tolerability, behavioral outcomes, and potential disease‐modifying effects were evaluated. Additional experiments investigated the influence of sigma‐1 antagonism (NE‐100) and genetic sigma‐1 deficiency on E1R efficacy and seizure development.

**Results:**

E1R delayed kindling acquisition, increased seizure thresholds in both naïve and kindled animals, and reduced seizure duration without evidence of tolerance or significant adverse effects. Subchronic E1R exposure slowed or prevented progression to generalized seizures, although effects did not persist after drug withdrawal. Sigma‐1 deficiency prolonged seizure duration, supporting a role in the termination of endogenous seizures. High‐dose NE‐100 partially antagonized E1R effects, whereas genetic deficiency did not, possibly due to compensatory mechanisms. Fenfluramine did not affect kindling progression in this model.

**Significance:**

Positive allosteric modulation of sigma‐1 attenuates ictogenesis and seizure severity and appears to contribute to endogenous seizure‐termination mechanisms. Although E1R influences seizure generation in response to repeated stimulation, it does not produce sustained disease‐modifying effects after discontinuation. These findings support a functional role of sigma‐1 positive allosteric modulators as promising candidates for seizure management in the model used. The absence of persistent effects after withdrawal argues against disease modification under the current conditions and warrants further investigation in chronic epilepsy models evaluating long‐term therapeutic, disease‐modifying, or preventive potential.

**Key points**

- E1R delayed progression in a mouse amygdala kindling model.
- NE‐100 partially antagonized the effects of E1R during kindling acquisition.
- Fenfluramine had only limited effects on kindling progression.
- Genetic deficiency of sigma‐1 prolonged seizure duration, supporting a role for endogenous sigma‐1 signaling in seizure termination.

## 1 INTRODUCTION

Sigma‐1 plays a crucial role in maintaining and restoring cellular homeostasis.^1^, ^2^ It can modulate the function of various receptors and ion channels, influencing mitochondrial function and the formation of reactive oxygen species.^2^, ^3^, ^4^, ^5^ Increasing knowledge of its essential chaperone function has inspired efforts to investigate sigma‐1 as a potential target for various neurological disorders, including epilepsy.^1^, ^3^, ^6^, ^7^, ^8^ In epileptology, interest in sigma‐1 was further piqued by evidence that the antiseizure medication fenfluramine may be a multitarget drug that not only influences serotonergic neurotransmission but also interacts with sigma‐1.^9^, ^10^, ^11^ However, the functional relevance of this interaction remains largely unexplored.

First studies confirmed antiseizure effects of sigma‐1 positive modulators in both acute and chronic seizure models with chemical seizure induction.^8^, ^12^, ^13^, ^14^ More recently, our study demonstrated relevant effects of the sigma‐1 positive allosteric modulator (4R,5S)‐2‐(5‐methyl‐2‐oxo‐4‐phenyl‐pyrrolidin‐1‐yl)‐acetamide (E1R) on ictogenesis in a chronic electrical kindling paradigm.^6^ The kindling paradigm is based on repeated electrical induction of seizures. Moreover, it avoids any bias from chemoconvulsants and enables a precise, controlled study of effects on the development of seizure parameters. Regarding the role of sigma‐1 as one target of fenfluramine, the findings were inconclusive, as we only found evidence in the kindling paradigm that the relatively minor effects of fenfluramine were limited by a sigma‐1 antagonist.^6^ Thus, further studies are of interest to determine the relevance of sigma‐1 targeting for the antiseizure medication fenfluramine.

Based on this context, we hypothesized that targeting sigma‐1 could not only have antiseizure effects but may also exert disease‐modifying and preventive effects.

Therefore, in this initial study, we evaluated the effects of the sigma‐1 positive modulator E1R and of fenfluramine when administered during kindling acquisition. The influence of a sigma‐1 antagonist and a genetic sigma‐1 deficiency on the pharmacological effects of E1R and fenfluramine was explored. In addition, we analyzed the impact of genetic sigma‐1 deficiency on the pharmacological antiseizure effects of E1R in fully kindled mice with increased seizure susceptibility.

## 2 MATERIALS AND METHODS

### 2.1 Animals

Female HsdWin:NMRI (*n* = 126, 24.2–31.7 g) and female and male ICR (CD‐1) wild‐type (WT) mice (*n* = 44, 27.4–45.0 g) were purchased from Inotiv. Homozygous breeding pairs of sigma‐1 knockout (Sig1R‐KO) mice (CD‐1.129‐Sigmar1tm1Lmon/Cnbc, *n* = 43, 30.8–46.1 g^15^) were provided by Welab and subsequently bred at our institute for the experiments. At the time of surgery, mice were 9–12 weeks old. All animals were housed under standardized conditions (22 ± 2°C, 55 ± 10% humidity, and a 12‐h light/dark cycle) with ad libitum access to tap water and standard laboratory food. Detailed information is provided in the Supporting Information. The experiments were conducted in line with the German Animal Welfare Act and EU Directive 2010/63/EU. The data are reported in accordance with the ARRIVE (Animal Research: Reporting of In Vivo Experiments) Guidelines 2.0 and the Basel Declaration, including the 3R principles. All studies were approved by the government of Upper Bavaria (license numbers: ROB‐55.2‐2532.Vet_02‐20‐99 and _02‐22‐44).

### 2.2 Surgery and electrode implantation

As described previously,^6^ the animals underwent stereotactic electrode implantation into the right amygdala for electrical stimulation and electroencephalographic recordings. Details on the perioperative multimodal analgesia, applied anesthesia, and surgical procedure are described in the Supporting Information.

### 2.3 Experimental design

**FIGURE 1:**
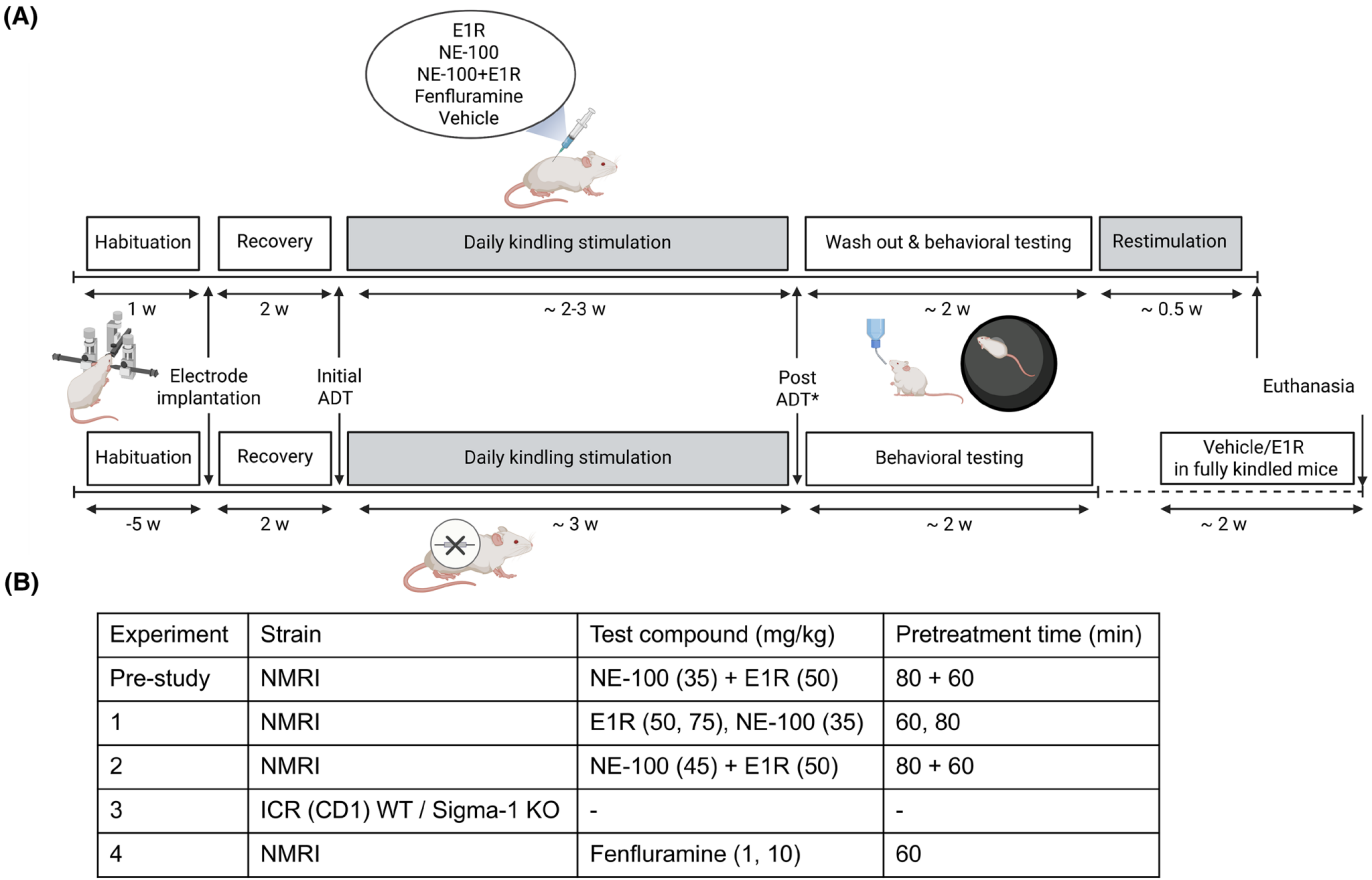
Experimental design and group allocation, showing schematic timeline of the study design (A) and tabular overview of the experimental groups, including mouse strain, test compound, and pretreatment time (B). ADT, afterdischarge threshold; w, week. Created in BioRender, von Rüden E (2026), https://BioRender.com/65cuw91.

This study comprised an initial prestudy and four subsequent main experiments (experiments 1–4). An overview of the experimental design and the allocation to the experimental and treatment groups is presented in Figure 1. NMRI mice were used in the experiments to analyze the impact of the test compounds on kindling progression: E1R and NE‐100 (experiment 1), cotreatment with NE‐100 and E1R (experiment 2), and fenfluramine (experiment 4). WT and Sig1R‐KO mice were used in experiment 3 to analyze the impact of genetic sigma‐1 deficiency. Vehicle‐treated kindled animals were used as controls for all experiments. Sham‐stimulated animals served as additional controls for experiments 1, 3, and 4. In accordance with the 3R principle, the same sham‐stimulated animals were used in experiments 1 and 4.

At the end of the experiments, animals were euthanized and transcardially perfused. Detailed information on euthanasia, transcardial perfusion, and determination of electrode localization is provided in the Supporting Information.

### 2.4 Test compounds

The following compounds were used for the experiments: the positive allosteric sigma‐1 modulator (4R,5S)‐2‐(5‐methyl‐2‐oxo‐4‐phenyl‐pyrrolidin‐1‐yl)‐acetamide (E1R, Latvian Institute of Organic Synthesis)^16^; the sigma‐1 antagonist 4‐methoxy‐3‐(2‐phenyl ethoxy)‐N, N‐dipropylbenzeneethanamine hydrochloride (NE‐100, Tocris/Bio‐Techne); the antiseizure medication fenfluramine (Zogenix, USA/UCB).

E1R was administered in doses of 50 and 75 mg/kg during kindling acquisition, and at 10, 25, 35, 50, and 75 mg/kg in fully kindled WT and Sig1R‐KO mice, with a pretreatment time (PTT) of 60 min for all doses. For NE‐100, a dose of 35 mg/kg was used in the prestudy. The dosing was subsequently increased to 45 mg/kg. The PTT for NE‐100 was 80 min in all experiments. Based on results from Perez‐Perez et al.,^6^ fenfluramine was administered at doses of 1 and 10 mg/kg with a PTT of 60 min. All compounds were dissolved in .9% saline (vehicle) and administered intraperitoneally.

### 2.5 Amygdala kindling

After determining the initial afterdischarge threshold (ADT), animals were allocated to different treatment groups by stratified randomization. The experimenter was unaware of the group allocation during kindling acquisition and behavioral testing.

Two to three days later, the impact of the test compounds on the prekindling ADT and on seizure parameters at threshold stimulation was assessed. Subsequently, the animals received once‐daily suprathreshold stimulations (Monday–Friday), with administration of either vehicle or test compound before the stimulation. The following parameters were recorded for each kindling stimulation: seizure severity (SS), seizure duration (SD), and afterdischarge duration 1 and 2 (ADD1 and ADD2; displayed as ADDT). The kindling process was terminated after 15 kindling stimulations, when most vehicle‐treated mice had developed at least 10 generalized seizures. Afterwards, the postkindling ADT was assessed.

Following the prestudy with an ineffective dose of NE‐100, the dose was increased in experiment 2 (cotreatment with E1R and NE‐100). In this experiment, we observed signs of reduced activity, increased muscle tone, tightened abdominal wall, and ataxia in most of the animals, which were suspected to be associated with repeated NE‐100 treatment. Moreover, two animals presented seizurelike behavior. Therefore, the kindling process was terminated after 10 kindling stimulations without post‐ADT determination.

Please refer to the Supporting Information for detailed definitions of the parameters and procedures involved.

In experiments 1, 3, and 4, following completion of the kindling process, behavioral patterns of the animals were assessed using the saccharin preference test and an open‐field paradigm. Detailed information on the behavioral tests and their results is provided in the Supporting Information.

### 2.6 Vehicle‐drug experiments in fully kindled WT and Sig1R‐KO mice

In an earlier study, we demonstrated that E1R exerts dose‐dependent antiseizure effects in fully kindled mice.^6^ To confirm whether these effects are sigma‐1 mediated, fully kindled WT and Sig1R‐KO mice were treated on alternating days with vehicle or E1R. On all experimental days, the ADT, generalized seizure threshold, and seizure parameters (SS, SD, and ADDT) were evaluated. Detailed information on the procedure and results is provided in the Supporting Information.

### 2.7 Statistics

The sample size was calculated using power analysis based on the parameter that resulted in the largest estimated sample size, ensuring that all analyses retained sufficient statistical power (GPower 3.1, Heinrich Heine University).^17^ Statistical analysis was performed using GraphPad Prism (version 10) and R 4.5.0 for Windows. All data are illustrated as mean ± standard deviation, and *p*‐values ≤ .05 were considered statistically significant. Detailed information on sample size calculation, randomization, and statistical analysis is provided in the Supporting Information.

## 3 RESULTS

### 3.1 Effects of E1R and NE‐100 on kindling progression

Pretreatment times and doses for E1R and NE‐100 were chosen based on earlier findings and recently published data from our group.^6^ The highest dose of E1R significantly increased the initial ADT (E1R_75_ *p* = .0086; Figure S2A). In contrast, neither a lower dose of E1R nor NE‐100 had a relevant effect. Seizure severity, behavioral seizure duration, and total afterdischarge duration remained largely unaffected by either of the two substances. An exception was 50 mg/kg E1R, which caused a slight increase in seizure severity (E1R_50_ *p* = .0313; Figure S2B–D).

**FIGURE 2:**
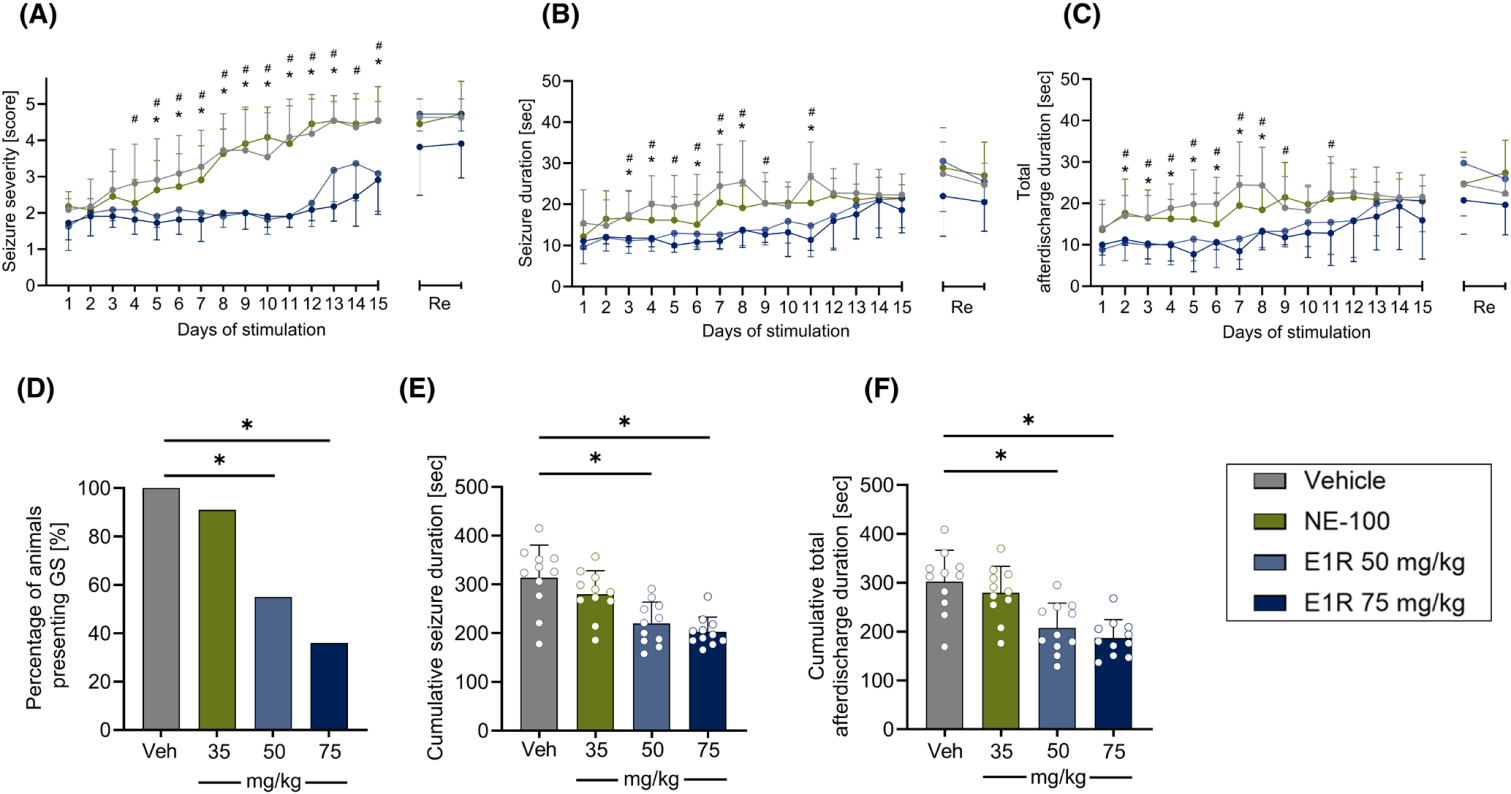
Effects of E1R (50 and 75 mg/kg) and NE‐100 (35 mg/kg) on kindling progression. E1R at both doses delayed the onset of generalized seizures (A) and transiently reduced the durations of motor and electroencephalographic seizures (B, C). NE‐100 did not affect seizure severity (A), seizure duration (B), or total afterdischarge duration (C). E1R treatment at both doses reduced the percentage of animals presenting generalized seizures (GS; D), the cumulative seizure duration (E), and the cumulative total afterdischarge duration (F), whereas NE‐100 treatment did not affect any of the analyzed parameters. Pretreatment times: E1R and vehicle, 60 min; NE‐100, 80 min. Please note, in accordance with the 3R principles, only one vehicle‐treated group was used as control for E1R and NE‐100. All data are presented as mean ± standard deviation. */#*p* ≤ .05, significant difference versus vehicle for E1R 50 or 75 mg/kg, respectively. Animal number in A–F: *n* = 11. Statistical tests: two‐way analysis of variance (ANOVA) with Dunnett post hoc test (A–C), Fisher exact test (D), and one‐way ANOVA with Dunnett post hoc test (E, F). Re, restimulation; Veh, vehicle.

During kindling progression, E1R delayed the onset of generalized seizures (treatment *p* < .0001, time *p* < .0001; Figure 2A). In addition, the duration of motor and electroencephalographic seizures was reduced at both doses (treatment *p* < .0001, time *p* < .0001 each; Figure 2B,C). Whereas this effect of E1R was evident during the early and middle stimulation phase, it was lost during the final stimulation phase. As expected, NE‐100 did not affect kindling acquisition (Figure 2A–D). The impact of E1R on kindling development is further supported by a significant decrease in the proportion of animals that reached generalized seizure stages at the end of the kindling phase (E1R_50_ *p* = .0351, E1R_75_ *p* = .0039; Figure 2D). Moreover, cumulative seizure duration (E1R_50_ *p* = .0002, E1R_75_ *p* < .0001; Figure 2E) and cumulative total afterdischarge duration (E1R_50_ *p* = .0004, E1R_75_ *p* < .0001; Figure 2F) proved to be reduced by E1R treatment at both doses.

Restimulation following washout of the compounds did not confirm robust and long‐lasting effects of the positive allosteric modulator E1R.

After discontinuation of E1R at 50 mg/kg, post‐ADT levels decreased (E1R_50_ *p* = .0086; Figure S3A). Additionally, regardless of the dose, discontinuation of E1R resulted in an increase in seizure severity (E1R_50_ *p* = .0039, E1R_75_ *p* = .0156; Figure S3B), whereas motor seizure and total afterdischarge duration remained unaffected (Figure S3C,D).

### 3.2 Impact of sigma‐1 antagonism with NE‐100 on E1R effects

Because the sigma‐1 antagonist NE‐100 at 35 mg/kg could not counteract the effects of E1R on kindling acquisition in the prestudy (Figures S4–S6), the dose of NE‐100 was increased to 45 mg/kg. As adverse effects had already been observed at higher doses in a previous study,^6^ the animals were monitored intensively using a standardized protocol. Initial ADTs were significantly increased (*p* = .0189) following combined treatment, with one drug‐exposed animal not reaching a measurable threshold. All other seizure parameters at threshold stimulation remained unaffected following combined exposure (Figure S7A–D).

**FIGURE 3:**
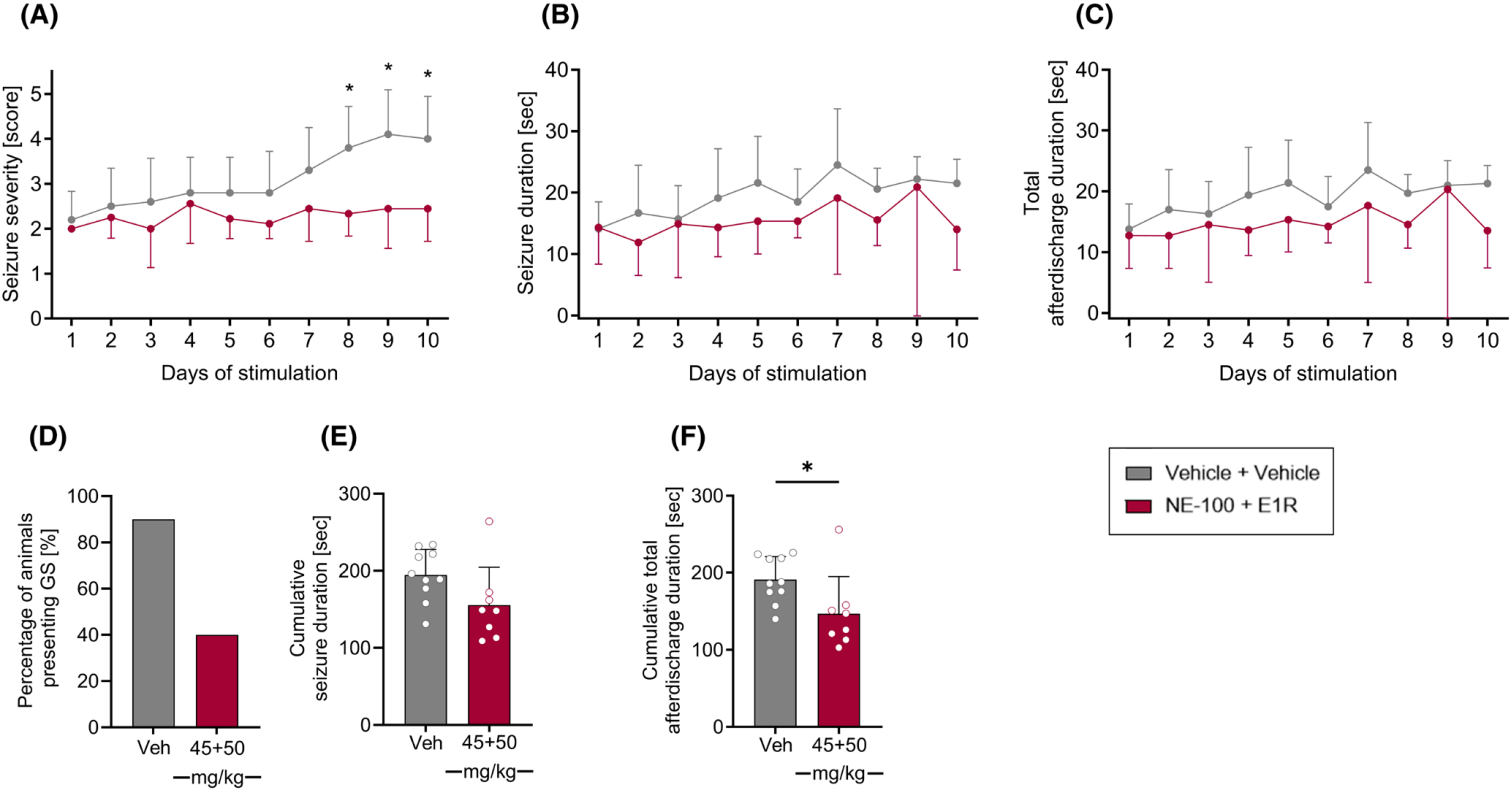
Effects of cotreatment with NE‐100 (45 mg/kg) and E1R (50 mg/kg) on kindling progression. The E1R effects were only partially prevented by NE‐100 cotreatment during the early kindling phase (A). This resulted in reduced seizure severity scores from stimulation day 8 onward. Seizure duration (B) and total afterdischarge duration (C) reached comparable levels in both treatment groups. Cotreatment with NE‐100 and E1R had no effect on the percentage of animals presenting generalized seizures (D) or on the cumulative seizure duration (E), whereas the cumulative total afterdischarge duration was reduced following cotreatment with NE‐100 and E1R (F). Please note that NE‐100 (45 mg/kg) caused tolerability issues; therefore, the stimulation phase was terminated after 10 stimulations. Pretreatment times: NE‐100, 80 min; E1R, 60 min; double vehicle at 80 min and 60 min. All data are presented as mean ± standard deviation. *p ≤ .05 versus vehicle. Animal numbers: (A–D), *n* = 9–10; (E, F), *n* = 8–10. One animal from the cotreatment group was excluded in panels E and F, due to missing values at stimulation day 2. Statistical tests: two‐way analysis of variance followed by Bonferroni post hoc test (A–C), Fisher exact test (D), and unpaired Student *t*‐test (E, F). Please note that due to the adverse effects of NE‐100 (45 mg/kg), the expected sample of *n* = 11 was not achieved for this experiment. GS, generalized seizures; Veh, vehicle.

Whereas NE‐100 prevented effects of E1R on seizure severity during the early stimulation phase, seizure severity was significantly reduced in drug‐exposed mice from stimulation day 8 onward (time *p* = .0002, treatment *p* = .0063; Figure 3A). Although E1R showed a significant overall effect on motor seizure and total afterdischarge duration (treatment *p*_SD_ = .0493 and *p*_ADDT_ = .0318), coadministration with the sigma‐1 antagonist prevented significant effects on individual stimulation days, as evident in the post hoc analysis (Figure 3B,C).

Following combined treatment with NE‐100 and E1R, the number of animals presenting generalized seizure activity during the kindling process and the cumulative seizure duration were not significantly different from those of animals with vehicle treatment (Figure 3D,E). In contrast, the cumulative total afterdischarge duration was significantly affected by drug exposure despite pretreatment with NE‐100 (*p* = .0301; Figure 3F).

### 3.3 Impact of genetic sigma‐1 deficiency on kindling progression

**FIGURE 4:**
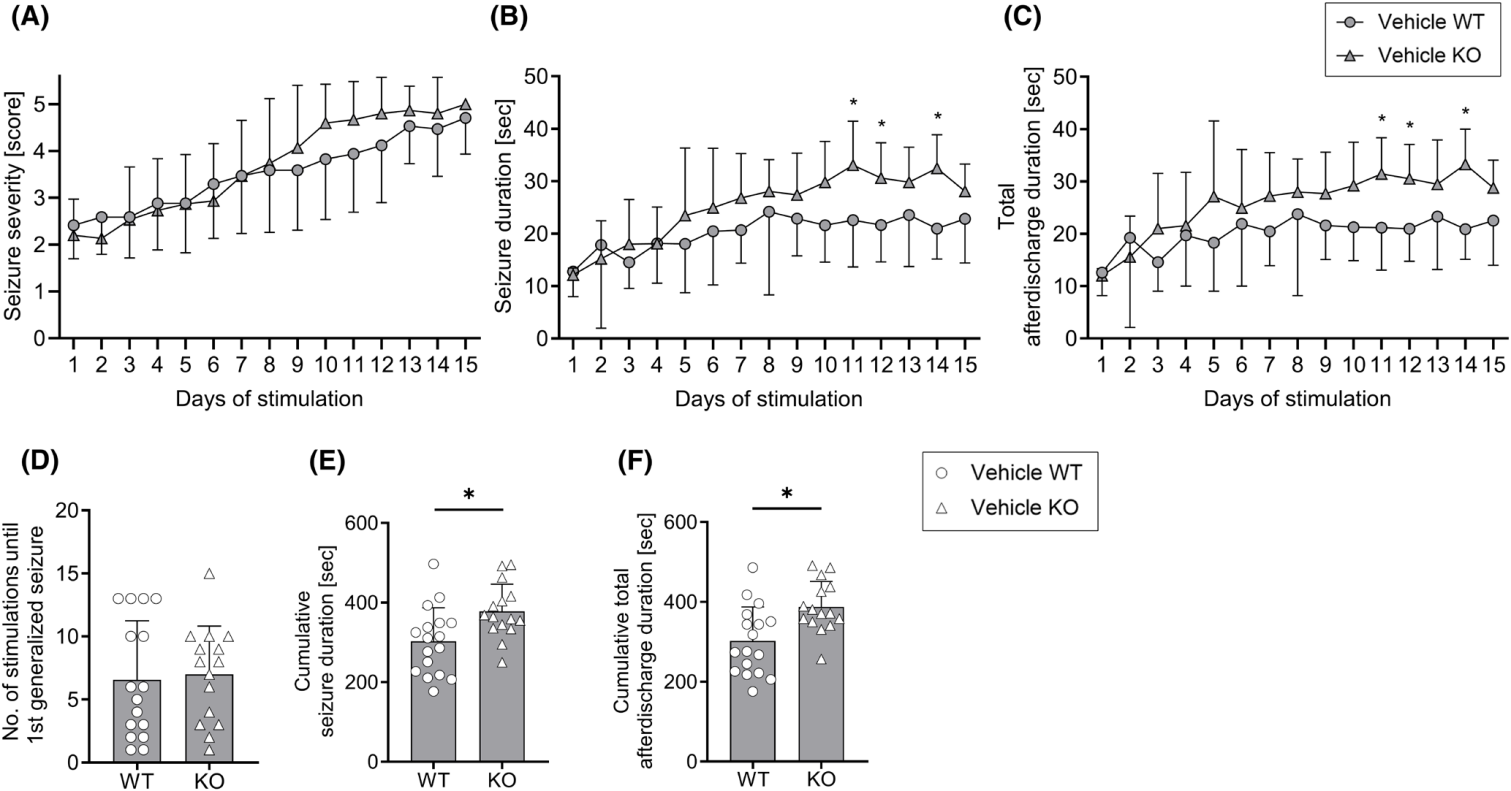
Impact of genetic sigma‐1 modulation on kindling progression in ICR (CD‐1) wildtype (WT) and sigma‐1 knockout (Sig1R‐KO) mice. Seizure severity during kindling acquisition is unaffected by genetic deficiency of sigma‐1 (A, D). In contrast, Sig1R‐KO mice exhibited longer behavioral and electroencephalographic seizures compared to WT mice (B, C). This is also reflected by a prolonged cumulative seizure duration (E) and cumulative total afterdischarge duration in Sig1R‐KO mice. All data are presented as mean ± standard deviation. *p ≤ .05 versus WT. Animal numbers: *n* = 15–17. One animal from the WT group did not develop generalized seizures and was therefore excluded from the data analysis in panel D. Statistical tests: two‐way analysis of variance followed by Bonferroni post hoc test (A–C), Mann–Whitney test (D), and unpaired Student *t*‐test with Welch correction (E, F). Please note that (aiming to reduce animal numbers following the 3R principle) all animals (WT and Sig1R‐KO) received vehicle treatment, as they served as controls in the experiment of E1R treatment in WT and Sig1R‐KO mice. These data can be found in the Supporting Information and Figures S6 and S7.

Genetic deficiency of sigma‐1 had no robust impact on the development of seizure severity during kindling acquisition (Figure 4A,D). In contrast, Sig1R‐KO mice exhibited prolonged behavioral or electroencephalographic seizure activity (treatment *p*_SD_ = .0101 and *p*_ADDT_ = .0035, respectively, and time *p* < .0001 each), with post hoc analysis revealing significant differences on single stimulation days during the late phase of kindling (Figure 4B,C). These findings are further supported by prolonged cumulative seizure and total afterdischarge durations in Sig1R‐KO mice (*p* = .0101 and *p* = .0035, respectively; Figure 4E,F).

To determine whether the genetic modulation has an influence on the effects of E1R, WT and Sig1R‐KO mice were treated with 50 mg/kg E1R. In both genotypes, E1R treatment resulted in a significant overall increase in prekindling ADT (treatment *p* = .0033); however, post hoc analysis revealed significant differences only in WT mice (*p* = .0375; Figure S8A). At threshold stimulation, all other parameters (i.e., seizure severity, motor, and total afterdischarge duration) remained unaffected by E1R in both genotypes (Figure S8B–D).

E1R delayed the onset of generalized seizures during kindling acquisition in both WT and Sig1R‐KO mice (time *p* < .0001, treatment *p* < .0001; Figure S9A). Consequently, fewer E1R‐treated mice reached generalized seizure stages compared to vehicle‐treated controls (*p*_WT_ = .0247, *p*_KO_ = .0169; Figure S9D). At several stimulation days, seizure duration (time *p* < .0001, treatment *p* < .0001; Figure S9B) and total afterdischarge duration (time *p* < .0001, treatment *p* < .0001; Figure S9C) proved to be lower in E1R‐treated WT and Sig1R‐KO mice. In line with this finding, a reduced cumulative seizure duration (*p*_WT_ = .008, *p*_KO_ < .0001; Figure S9E) and afterdischarge duration (*p*_WT_ = .0072, *p*_KO_ < .0001; Figure S9F) became evident in WT and Sig1R‐KO mice.

Discontinuation of E1R resulted in a drop of the postkindling ADT in both WT and Sig1R‐KO mice (*p*_WT_ = .0157, *p*_KO_ = .0041; Figure S10A) and an increase in seizure severity in WT mice (*p* = .031; Figure S10B). These changes are associated with an increase in seizure and total afterdischarge durations in WT mice (*p*_SD_ = .0217, *p*_ADDT_ = .0354; Figure S10C,D).

### 3.4 Effects of fenfluramine on kindling progression

**FIGURE 5:**
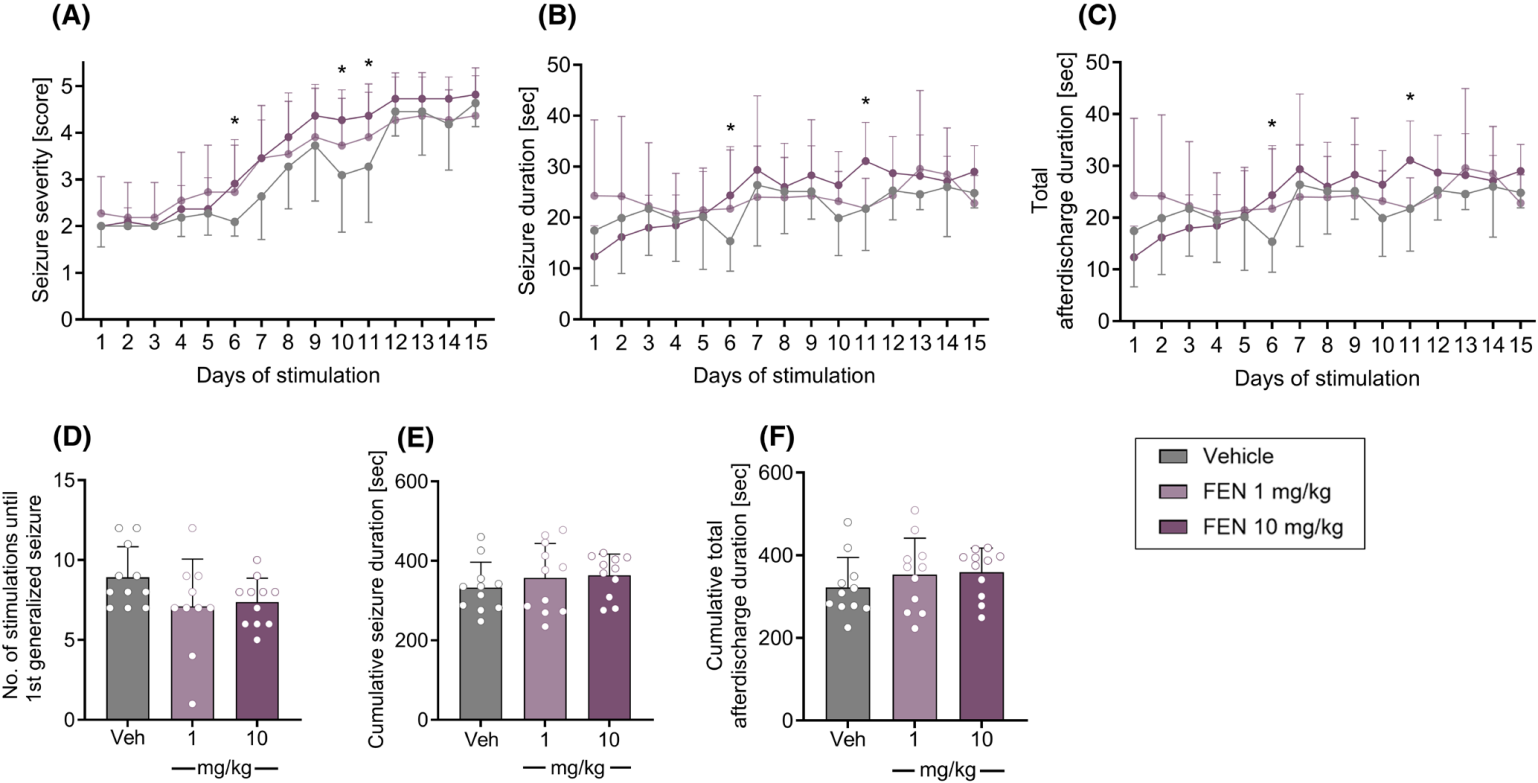
Effects of fenfluramine (FEN; 1 and 10 mg/kg) on kindling progression. FEN had only limited effects on seizure severity (A), seizure duration (B), and total afterdischarge duration (C), with treated mice (10 mg/kg) showing higher seizure scores and seizure durations on individual stimulation days. FEN treatment did not impact the number of stimulations required to induce the first generalized seizure (D) nor the cumulative seizure duration (E) or the cumulative total afterdischarge duration (F) when compared to vehicle (Veh)‐treated animals. Pretreatment time: 60 min. All data are presented as mean ± standard deviation. *p ≤ .05, significant difference versus vehicle for FEN 10 mg/kg. Animal numbers: *n* = 11. One animal from the FEN group (1 mg/kg) did not develop generalized seizures and was therefore excluded from the data analysis in panel D. Statistical tests: two‐way analysis of variance (ANOVA) followed by Dunnett post hoc test (A–C), Kruskal–Wallis test (D), and one‐way ANOVA (E, F).

Fenfluramine was tested at doses of 1 and 10 mg/kg based on earlier findings in fully kindled mice.^6^ Neither dose affected initial ADTs and seizure parameters at threshold stimulation (Figure S13A–D). During kindling acquisition, there was no overall treatment effect; however, a significant time × treatment interaction was observed (*p*_SS_ < .0001, *p*_SD_ = .0171, time *p*_ADDT_ = .0171; Figure 5A–C), and post hoc analysis revealed that seizure severity as well as motor and electroencephalographic seizure durations reached higher levels in fenfluramine‐treated mice (10 mg/kg) at single stimulation days as compared to vehicle‐treated mice (Figure 5A–C). However, analyses of the number of stimulations required to reach the first generalized seizure, as well as cumulative motor seizure and total afterdischarge durations, did not confirm robust effects of fenfluramine on the overall kindling rate (Figure 5D–F). Postkindling ADTs remained unaffected following drug discontinuation (Figure S14E–H).

**FIGURE 6:**
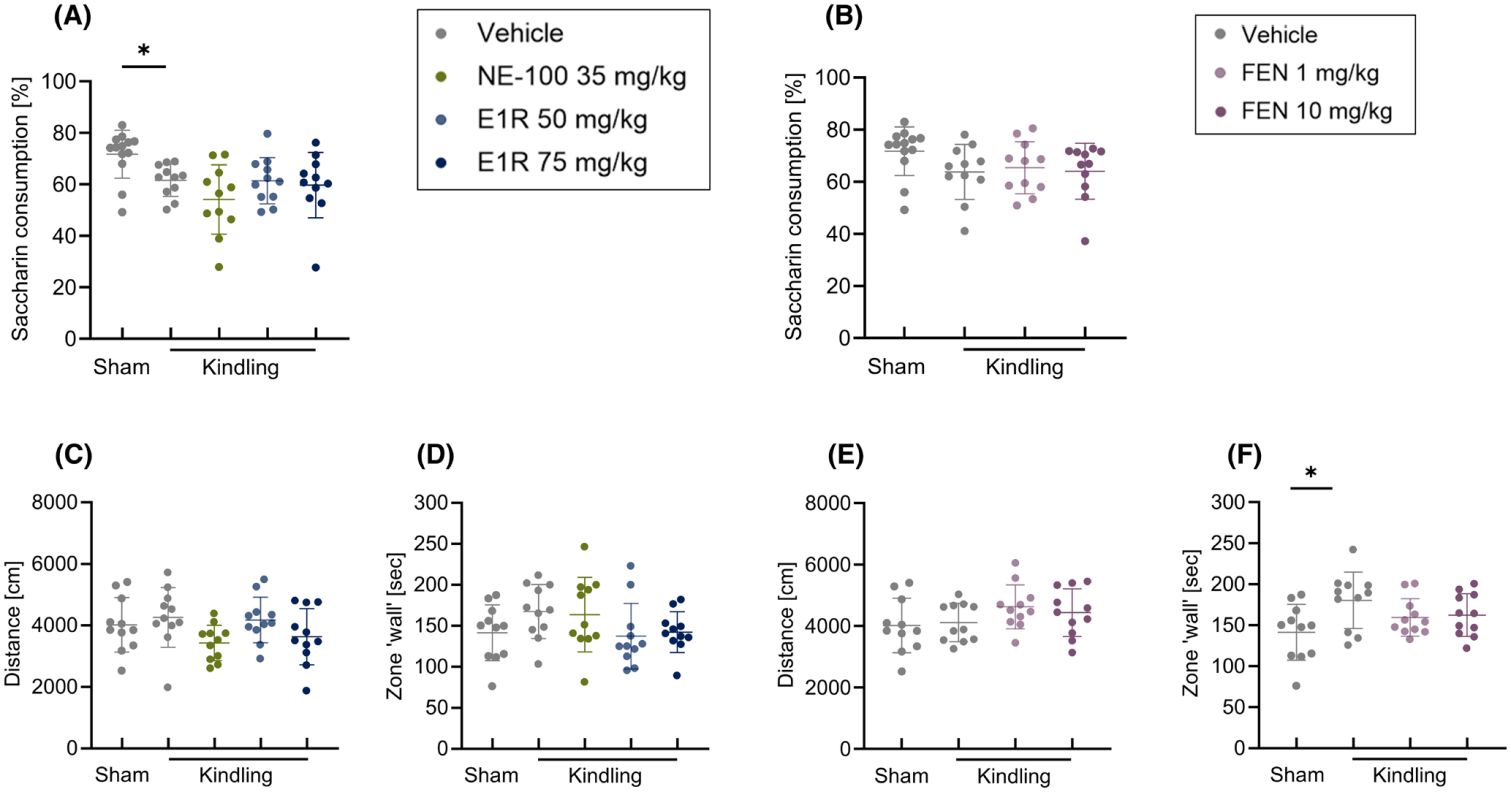
Effects of pharmacological sigma‐1 modulation on saccharin consumption (A, B) and on behavioral parameters in the open‐field test during the first 5 min (total time: 15 min; C–F). Kindling reduced saccharin preference, whereas neither of the drugs, NE‐100 nor E1R, reversed this effect (A). The distance moved remained unaffected by the kindling process (C, E). During the exploratory phase, a kindling effect was evident in fenfluramine (FEN) experiments, with kindled animals spending more time in the wall zone (D, F). Pharmacological treatment with FEN could not mitigate this effect. Please note, in accordance with the 3R principles, the same sham‐stimulated animals were used in experiments 1 and 4. All data are presented as mean ± standard deviation. *p ≤ .05 versus sham‐stimulated animals. Animal numbers: *n* = 11–13. Statistical tests: unpaired *t*‐test (A–F) followed by one‐way analysis of variance (A, F).

Detailed information on the effects of pharmacological and genetic sigma‐1 modulation on anhedonia‐associated behavior, locomotion, and exploration is provided in the Supporting Information and Figure 6.

## 4 DISCUSSION

Due to its important homeostatic function, sigma‐1 is considered a promising therapeutic target in the brain for various neurological indications.^1^, ^2^ Building on our recent findings,^6^ we aimed to further complete our understanding of the role of sigma‐1 in ictogenesis, seizure spread, and seizure termination. Moreover, we investigated whether targeting of sigma‐1 could have disease‐modifying and preventive effects beyond pure antiseizure effects. Our study, which used the amygdala kindling paradigm, provides new insights into the effects of a positive allosteric sigma‐1 modulator and the role of sigma‐1 on the development and progression of seizures.

The positive allosteric modulator E1R delayed the progression of kindled seizures, with a significant impact on the development of the severity and duration of electrically triggered seizure events. E1R was selected for these experiments because positive allosteric modulators offer a way to increase sigma‐1 activity in a use‐dependent manner, which is considered a superior approach in terms of tolerability.^18^ In contrast, the use of direct agonists would activate the chaperone protein sigma‐1 and its downstream signaling events, maintaining a persistent alert state. This can have detrimental implications for the tolerability of the targeting approach and can interfere with an on‐demand activation of the protective system in response to transient cellular stress. Supporting an advantageous profile of sigma‐1 positive allosteric modulators, E1R was well tolerated in the kindling paradigm, with no evidence of severe adverse effects. Interestingly, this was true for both naive animals at the start of the kindling paradigm and fully kindled mice at the end of the kindling acquisition phase. This finding is of relevance, as earlier studies have demonstrated an increased susceptibility to adverse effects of drug candidates, which translates to patients with epilepsy.^19^, ^20^, ^21^ It should be noted that the analysis in this study was limited to commonly used clinical scoring systems; therefore, more comprehensive assessments of tolerability in chronic epilepsy models are warranted in future studies.

Concerning the antiseizure effects of E1R, the findings from the current study are in line with our earlier results from fully kindled mice.^6^ However, our new findings also demonstrate that dose‐dependent antiseizure effects with an increase in seizure thresholds are not limited to animals with a hyperexcitable neuronal network resulting from the kindling procedure but are also evident in naive animals at the beginning of the procedure. Thus, positive allosteric modulators might be of interest not only for seizure management in patients with chronic epilepsy but also for therapeutic management in clinical scenarios with a transiently increased risk of acute seizures. This conclusion is further supported by earlier studies that explored the effects of E1R and other selective positive allosteric modulators in acute models with chemical induction of seizures in naive animals.^8^, ^12^, ^14^ However, interpretation of these data requires caution, as chemoconvulsants directly interact with receptors and ion channels that are modulated by sigma‐1.

As stated above, subchronic exposure to E1R significantly delayed kindling progression and reduced the transition to more severe generalized seizures in response to daily stimulation. These findings suggest that positive allosteric modulation of sigma‐1 can interfere with the development of a hyperexcitable kindled network. To our knowledge, this is the first report of effects in a chronic paradigm with repeated seizure induction and subchronic exposure to E1R. However, this impact was only observed during ongoing treatment and did not persist after drug withdrawal of the test compound, arguing against a sustained disease‐modifying effect under the present conditions. Instead, the delayed progression may reflect the cumulative impact of antiseizure effects during daily kindling stimulations.

Future studies will be necessary to address the question of whether positive allosteric modulation of sigma‐1 can exert actual disease‐modifying and preventive effects in chronic epilepsy models with the development of spontaneous recurrent seizures. Nevertheless, to our knowledge, the data from the kindling study provide the first evidence that subchronic administration of E1R is associated with lasting effects on ictogenesis without the development of tolerance. This finding is particularly relevant, as a reduction or loss of therapeutic effect is known to occur with the antiseizure effects of benzodiazepines and, in certain patient subgroups, also with other selected antiseizure medications such as levetiracetam.^22^, ^23^, ^24^

The finding that E1R shortened seizure duration throughout the course of the kindling procedure could indicate a role of sigma‐1 in the endogenous termination of seizures. Alternatively, the influence on duration could also be indirect, as less severe focal seizures in the kindling model typically have a shorter duration than generalized seizures.

Analysis of behavioral patterns in the saccharin preference test and the open‐field paradigm did not provide evidence for a long‐lasting modification of nonseizure symptoms. In general, kindling‐associated alterations are relatively mild,^25^, ^26^ making it challenging to detect potential beneficial effects of the test compounds. Consistent with this, we did not observe any relevant behavioral changes in kindled mice following vehicle administration.

Various studies provided evidence that sigma‐1 antagonists can protect from convulsions associated with an overdose of psychostimulants or drugs of abuse.^8^, ^27^, ^28^, ^29^, ^30^, ^31^, ^32^ On the other hand, further investigations demonstrated that the sigma‐1 antagonist NE‐100 can lower seizure thresholds in an acute chemical seizure model, following the administration of the γ‐aminobutyric acid (GABA) antagonist pentylenetetrazole (PTZ), and can directly trigger ictogenesis, indicating proconvulsant effects at higher doses (50 and 75 mg/kg).^14^ Analysis in the kindling paradigm did not indicate a relevant impact of repeated administration of a lower dose of NE‐100 (35 mg/kg) on electrically induced seizures in this paradigm, arguing against a relevant proseizure effect of sigma‐1 antagonism. Thus, earlier findings with low doses of NE‐100 in the PTZ model might have been biased by the mechanism of action of the chemoconvulsant.

For direct comparison, we have additionally assessed the impact of sigma‐1 genetic deficiency on kindling acquisition. Although the respective analysis did not reveal a relevant influence of the lack of functional sigma‐1 on seizure severity, the cumulative duration of kindled seizures was increased, related to prolonged seizure events on selected stimulation days. This finding suggests a potential role of sigma‐1 in endogenous seizure termination. Immediate effects of sigma‐1 translocation to the membrane are conceivable due to the direct influence of sigma‐1 on the function of various glutamate and GABA receptors and voltage‐gated ion channels.^33^, ^34^, ^35^, ^36^, ^37^, ^38^ A key role of sigma‐1 in restoring the balance between inhibitory and excitatory neurotransmission, as well as in regulating the distribution of sodium, calcium, and potassium, has been reported in various pathophysiological scenarios associated with neurological disorders.^34^, ^39^, ^40^ Among other mechanisms, sigma‐1 appears to suppress N‐methyl‐D‐aspartate receptor‐mediated excitatory neurotransmission by modulating subunit trafficking and influencing the inactivation of voltage‐gated sodium channels, thereby reducing their firing rates.^35^, ^36^, ^41^, ^42^ Loss of sigma‐1 could thus impair these as well as other sigma‐1‐associated mechanisms that contribute to the termination of seizures. In this context, it is also notable that Vavers et al.^43^ have linked genetic inactivation of sigma‐1 to reduced expression of the GABA‐B receptor subunit R2, which may also affect the termination of seizure activity. These mechanistic consequences of sigma‐1 activation or loss, together with our findings, indicate that future studies should investigate the therapeutic effects of positive allosteric sigma‐1 modulators on prolonged seizure events such as clusters or status epilepticus.

To assess the sigma‐1 dependency of E1R effects in the kindling paradigm, we investigated both the influence of the sigma‐1 antagonist NE‐100 and genetic sigma‐1 deficiency on E1R efficacy. Although a lower dose (35 mg/kg) failed to exert relevant effects, a higher dose of NE‐100 (45 mg/kg) partially antagonized the effects of E1R. Unfortunately, this dose was not well tolerated, with subchronic dosing resulting in an early termination of the experiment. In contrast, an acute dose of 45 mg/kg NE‐100 was better tolerated in an earlier study, in which 25, 35, and 45 mg/kg NE‐100 diminished the response of fully kindled mice to 50 mg/kg E1R.^6^ As the adverse effects became more apparent during the course of the experiment, repeated administration and subchronic exposure may have had a negative impact on the tolerability of NE‐100.

Surprisingly, sigma‐1 genetic deficiency did not efficaciously counteract the effects of E1R. This observation may be due to compensatory changes resulting from sigma‐1 deficiency and possible off‐target effects of E1R. However, the selectivity of E1R has been comprehensively characterized using commercial screening assays that test possible interactions with various neuronal receptors and ion channels.^16^

Fenfluramine has become an important component in the therapeutic management of the developmental and epileptic encephalopathies, Dravet syndrome and Lennox–Gastaut syndrome.^44^, ^45^ Despite this, the mechanism of action of fenfluramine is still incompletely understood.^46^ Although there is convincing evidence that the modulation of serotonergic neurotransmission, through direct interaction with various receptor subtypes and an impact on extracellular serotonin concentrations, is functionally relevant for the antiseizure effects of fenfluramine, the relevance of further mechanisms has not been studied in detail.^46^ Among these possible mechanisms, the interaction with sigma‐1 is particularly noteworthy, given the high affinity of fenfluramine to the sigma‐1 chaperone molecule.^10^ Findings from a mouse model of dizocilpine learning deficits,^10^, ^47^ in combination with earlier studies describing antiseizure effects of positive modulators, led the authors of subsequent reviews to suggest that sigma‐1 is likely to play a pivotal role as one of several targets of fenfluramine.^9^, ^46^ However, direct proof for this functional relevance in the context of fenfluramine's antiseizure potency is still lacking.

Here, we aimed to further explore the potential of fenfluramine for epilepsies with etiologies other than the developmental and epileptic encephalopathies, and in the case of a robust effect, to determine the relevant contribution of sigma‐1 to its antiseizure efficacy. As the corresponding experiment failed to detect relevant effects of fenfluramine during kindling acquisition, we did not continue with the combination experiments. Taken together with positive findings from an intrahippocampal kainate model and negative data from testing in fully kindled mice, the efforts to assess a broad‐spectrum potential point to a mixed efficacy profile in models of acquired temporal lobe epilepsy. The intrahippocampal kainate mouse model, as well as the amygdala kindling model, are well established and validated animal models of temporal lobe epilepsy. However, they capture distinct aspects of epileptic network pathology. The intrahippocampal kainate model reflects structural reorganization of the neuronal network driven by a chemical insult, resulting in spontaneous epileptiform activity, whereas kindling primarily represents stimulation‐driven seizure responses and progressive activity‐dependent neuronal and network plasticity. The observed discrepancy of fenfluramine effects may therefore be context‐ and endpoint‐dependent, with a stronger impact on spontaneous epileptiform activity. Moreover, given fenfluramine's multimodal mechanism, including serotonergic enhancement and modulation of the sigma‐1 protein, differential network states and receptor engagement across models may contribute to the observed effects. In addition, differences in biological background (genetic strain and sex) may have influenced serotonergic and sigma‐1‐related signaling, further contributing to model‐specific pharmacological responsiveness. Thus, further studies in other chronic models would be of interest. Regarding the interaction of fenfluramine with sigma‐1, studies combining fenfluramine with a sigma‐1 antagonist in a genetic Dravet mouse model could help clarify its relevance.

In conclusion, our findings demonstrate that positive allosteric modulation of sigma‐1 with E1R delays the progression of kindling, elevates seizure thresholds, and shortens seizure duration without inducing tolerance or major adverse effects. These findings support a functional role of sigma‐1 in ictogenesis, seizure propagation, and endogenous seizure termination. Although E1R influenced network hyperexcitability during repeated stimulation, the absence of persistent effects after withdrawal argues against disease modification under the current conditions. Further work in chronic epilepsy models is needed to determine whether sigma‐1 modulation can provide long‐lasting preventive or therapeutic benefits.

## AUTHOR CONTRIBUTIONS

**Eva‐Lotta von Rüden:** Methodology; formal analysis; data curation; writing—original draft; writing—review & editing; visualization; supervision; project administration; funding acquisition. **Verena Buchecker:** Methodology; formal analysis; supervision; project administration; writing—original draft; writing—review & editing; visualization. **Amelie Wagner:** Methodology; investigation; writing—review & editing. **Victoria Stocker:** Methodology; investigation; writing—original draft; writing—review & editing. **Liga Zvejniece:** Conceptualization; resources; writing—review & editing. **Heidrun Potschka:** Conceptualization; methodology; resources; writing—original draft; writing—review & editing; supervision; project administration; funding acquisition.

## FUNDING INFORMATION

DFG (PO 681/12‐1) and European Cooperation in Science and Technology (COST Association), SIGMA‐1_EUROPE action, No. CA23156.

## CONFLICT OF INTEREST STATEMENT

H.P. has served as a paid consultant for Zogenix. The remaining authors have no conflicts of interest. We confirm that we have read the Journal's position on issues involved in ethical publication and affirm that this report is consistent with those guidelines.

## Supporting information


Appendix S1.

Data S1.

## Data Availability

Data are available through Figshare (https://doi.org/10.6084/m9.figshare.32006313).
