# Supplementary material for: Effects of fenfluramine and sigma‐1‐dependent pharmacological and genetic modulation in a mouse kindling model
Source: Epilepsia. 2026 Apr 29;67(8):4292–304. doi: 10.1002/epi.70270 (PMC13525583; doi:10.1002/epi.70270)
Supplement: Supplementary file 1 [file EPI-67-4292-s001.docx]

**Effects of Fenfluramine and Sigma-1-dependent pharmacological and genetic modulation in a mouse kindling model**

Eva-Lotta von Rüden*^1^, Verena Buchecker*^1^, Amelie Wagner^1^, Victoria M. Stocker^1^, Liga Zvejniece^2^ and Heidrun Potschka^1^

*Both authors contributed equally

**Supplementary figures**

Figure S1. Diagram with detailed information on exclusion or loss of animals during the course of the experiments.

Figure S2. (A-D) Effects of E1R (50 and 75 mg/kg) and NE-100 (35 mg/kg) on initial afterdischarge thresholds (ADT) and seizure parameters at threshold stimulation. E1R at 75 mg/kg increased the delta (Δ) initial ADT (A), while seizure severity (B), behavioral seizure duration (C), and total afterdischarge duration (D) remained largely unaffected. A slight increase in seizure severity was only observed at 50 mg/kg E1R (B). The delta change represents the difference between the initial ADT determined in the untreated condition and the initial ADT after treatment with vehicle, NE-100, or E1R. All data are presented as mean ±SD. *p≤0.05 versus vehicle. Animal numbers: n = 11. Two animals treated with E1R 75 mg/kg did not develop an ADT and were therefore excluded from the respective dose-specific analysis shown in panels A-D. Statistical tests: One-way ANOVA followed by Dunnett’s post hoc test (A, C, and D) and Wilcoxon matched-pairs signed rank test for seizure severity (B).

Figure S3. (A-D) Effects of discontinuation of E1R (50 and 75 mg/kg) and NE-100 (35 mg/kg) on post-afterdischarge threshold (ADT) and seizure parameters at threshold stimulation. Discontinuation of E1R resulted in decreased post-ADT levels at 50 mg/kg (A) and in increased seizure severity scores at both dosages (B). Seizure duration was significantly reduced following discontinuation of vehicle treatment (C). No significant effects were observed for the total afterdischarge duration (D). All data are presented as mean ± SD. *p≤0.05. Animal numbers: n = 11. Statistical tests: paired Student’s t-test (A, C, and D) and Wilcoxon matched-pairs signed rank test for seizure severity (B).

Figure S4. (A-D) Pre-study: Effects of co-administration of NE-100 (35 mg/kg) and E1R (50 mg/kg) on initial afterdischarge thresholds (ADT) and seizure parameters at threshold stimulation. Co-administration of NE-100 and E1R resulted in a significant increase in delta (Δ) initial ADT compared to vehicle-treated mice (A). All other seizure parameters at threshold stimulation remained unaffected (B–D). The delta change represents the difference between the untreated condition and after treatment with either vehicle or NE-100 and E1R. All data are presented as mean ± SD. *p≤0.05 versus vehicle. Animal numbers: n = 9-10. Statistical tests: unpaired Student’s t-test (A, C, and D) and Wilcoxon matched-pairs signed rank test for seizure severity (B). Please note that the expected sample size of n = 11 was not achieved for this experiment.

Figure S5. (A-F) Pre-study: Effects of co-administration of NE-100 (35 mg/kg) and E1R (50 mg/kg) on kindling progression. Despite co-administration of NE-100, E1R delayed the progression of kindling, leading to lower seizure severity scores (A), affecting seizure (B), and total afterdischarge duration (C) on individual days. In addition, co-administration decreased the proportion of animals that developed generalized seizures at the end of the kindling process (D) and reduced the cumulative seizure duration (E) and total afterdischarge (F) duration. All data are presented as mean ± SD. *p≤0.05 versus vehicle. Animal numbers: n = 10-11. Statistical tests: two-way ANOVA with Bonferroni post hoc test (A-C), Fisher’s exact test (D), and unpaired Student’s t-test (E and F). Please note that the expected sample size of n = 11 was not achieved for this experiment.

Figure S6. (A-D) Pre-study: Effects of discontinuation of co-administration of NE-100 (35 mg/kg) and E1R (50 mg/kg) on post-afterdischarge threshold (ADT) and seizure parameters at threshold stimulation. Co-administration of NE-100 and E1R had no effect on seizure parameters compared to the post-ADT determined under untreated conditions. All data are presented as mean ± SD. Animal numbers: n = 9-10. Statistical tests: paired Student’s t-test (A, C, and D) and Wilcoxon matched-pairs signed rank test for seizure severity (B). Please note that the expected sample size of n = 11 was not achieved for this experiment.

Figure S7. (A-D) Effects of co-treatment with NE-100 at 45mg/kg and E1R at 50 mg/kg on initial afterdischarge thresholds (ADT) and seizure parameters at threshold stimulation. Co-administration of NE-100 at 45 mg/kg and E1R at 50 mg/kg resulted in a significant increase in delta (Δ) initial ADT compared to vehicle-treated mice (A). All other seizure parameters at threshold stimulation remained unaffected (B–D). The delta change represents the difference between the untreated condition and after treatment with vehicle or NE-100 and E1R. All data are presented as mean ± SD. *p≤0.05. Animal numbers: n = 8-10. Statistical tests: unpaired Student’s t-test (A) with Welch’s correction (C and D) and Wilcoxon matched-pairs signed rank test for seizure severity (B). Please note, the kindling process was terminated after 10 stimulations, and post-ADTs were not determined due to side effects caused by NE-100 at 45mg/kg. The calculated sample size (n = 11) was not achieved for this experiment.

Figure S8. (A-D) Impact of vehicle and E1R (50 mg/kg) treatment on initial afterdischarge thresholds (ADT) in ICR (CD-1) wildtype (WT) and Sigma-1 knockout (Sig1R-KO) mice. Following treatment with E1R (50 mg/kg), WT mice showed increased initial ADTs (A). Seizure severity, motor, and total afterdischarge duration remained unaffected by E1R in both genotypes (B-D). The delta change represents the difference between the determination of the initial ADT in the untreated condition and after the treatment with vehicle or E1R. All data are presented as mean ± SD. *p≤0.05 versus vehicle. Animal numbers: n = 13-16. Statistical tests: two-way ANOVA with Bonferroni multiple comparison test (A, C, and D) and Wilcoxon matched-pairs signed rank test for seizure severity (B).

Figure S9. (A-F) Effects of treatment with vehicle or E1R at 50 mg/kg on kindling acquisition in ICR (CD-1) wildtype (WT) and Sigma-1 knockout (Sig1R-KO) mice. Treatment with E1R (50 mg/kg) resulted in a delayed onset of generalized seizures in both genotypes (A). In addition, treatment with E1R transiently decreased seizure duration and total afterdischarge duration in both genotypes compared to vehicle-treated mice (B-C). This is in line with fewer E1R-treated animals developing generalized seizures compared to vehicle-treated control animals (D). In both genotypes, E1R treatment resulted in a decreased cumulative seizure (E) and total afterdischarge duration (F). All data are presented as mean ± SD. *p≤0.05 versus vehicle. Animal numbers: n = 13-16. Statistical tests: three-way ANOVA and built-in two-way ANOVA with Bonferroni multiple comparison test (A-C), Fisher’s exact test (D), and two-way ANOVA with Bonferroni multiple comparison test (E and F).

Figure S10. (A-D) Impact of discontinuation of vehicle and E1R (50 mg/kg) treatment on post-kindling afterdischarge thresholds (ADT) in ICR (CD-1) wildtype (WT) and Sigma-1 knockout (Sig1R-KO) mice. Discontinuation of E1R resulted in reduced post-kindling ADTs in WT and Sig1R-KO mice (A), as well as in an increased seizure severity in WT mice (B). This is associated with an increase in seizure (C) and total afterdischarge duration (D) in Sig1R-KO mice. All data are presented as mean ± SD. *p≤0.05. Animal numbers: n = 13-16. Statistical tests: paired Student’s t-test (A, C, and D) and Wilcoxon matched-pairs signed rank test for seizure severity (B).

Figure S11. (A-D) Effect of E1R (pretreatment time of 60 minutes) on the afterdischarge thresholds (ADT) and seizure parameters at threshold stimulation in fully kindled ICR (CD-1) wildtype (WT) and Sigma-1 knockout (Sig1R-KO) mice. Whereas a lower dosage of 10 mg/kg slightly decreased the ADT in Sig1R-KO mice, higher doses of E1R (50 and 75 mg/kg) increased the ADT in WT mice. In Sig1R-KO mice, a drug effect was only evident at a dose of 75 mg/kg E1R (A). The seizure severity was reduced by E1R at 75 mg/kg in WT mice and by 25, 35, and 75 mg/kg E1R in Sig1R-KO mice and by 75 mg/kg in WT mice (B). Seizure and total afterdischarge duration are unaffected by treatment or genotype. All data are presented as mean ± SD. *p≤0.05 versus vehicle. Animal numbers: n = 13-16. Statistical tests: paired Student’s t-test (A, C and D) and Wilcoxon matched-pairs signed rank test (B).

Figure S12. (A-C) Effects of E1R (pretreatment time of 60 minutes) in fully kindled ICR (CD-1) wildtype (WT) and Sigma-1 knockout (Sig1R-KO) mice on generalized seizure thresholds (GST) and seizure parameters at threshold stimulation. An E1R-induced increase of the GST was observed independent of the genotype at dosages of 35, 50, and 75 mg/kg. At a lower dosage of 25 mg/kg the GST slightly increased in WT mice, but not in Sig1R-KO mice (A). Behavioral seizure (B) and electrographic seizure duration (C) were prolonged independent of the genotype in the higher dosage range (50 and 75 mg/kg E1R), whereas this effect was inconsistent in the lower dosage range (10, 25, and 35 mg/kg E1R). All data are presented as mean ± SD. *p≤0.05 versus vehicle. Animal numbers: n = 13-16. Statistical test: paired Student’s t-test.

Figure S13. (A-D) Impact of Fenfluramine (1 and 10 mg/kg) treatment on initial afterdischarge thresholds (ADT) and seizure parameters at threshold stimulation. At both doses, Fenfluramine had no effects on ADTs and seizure parameters at threshold stimulation. Animal numbers: n = 11. All data are presented as mean ± SD. Statistical tests: one-way ANOVA (A, C, and D) and Wilcoxon matched-pairs signed rank test (B).

Figure S14. (A-D) Impact of discontinuation of Fenfluramine (1 and 10 mg/kg) treatment on post-afterdischarge thresholds (ADT) and seizure parameters at threshold stimulation. Following discontinuation, only vehicle-treated animals showed reduced post-ADT values (A) and seizure durations (C). Seizure severity scores (B) and total afterdischarge remained unaffected. Animal numbers: n = 11. All data are presented as mean ± SD. *p≤0.05. Statistical tests: paired Student’s t-test (A, C, and D) and Wilcoxon matched-pairs signed rank test (B).

Figure S15. (A) Effects of genetic Sigma-1 modulation on saccharin consumption in ICR (CD-1) wildtype (WT) and Sigma-1 knockout (Sig1R-KO) mice. Vehicle-treated and kindled Sig1R-KO mice showed reduced saccharin consumption compared to both vehicle-treated and kindled WT mice and non-kindled sham Sig1R-KO animals. E1R was able to normalize the effects of genetic Sigma-1 deficiency. All data are presented as mean ± SD. *p≤0.05. Animal numbers: n = 13-17. Statistical test: two-way ANOVA with Bonferroni multiple comparison test.

Figure S16. Impact of pharmacological (A-F) and genetic (G-I) modulation of Sigma-1 on locomotor (A, D, G) and exploratory behavior in the open-field test over the total duration of 15 minutes. Across all experiments, the distance moved was unaffected by the kindling process (A, D, G). Kindling did not affect exploratory behavior (B, C, E, F, H, and I), but Sigma-1 knockout mice (Sig1R-KO) spent more time in the wall zone (H) and less time in the center zone (I) when compared to the respective WT group. All data are presented as individual data points and mean ± SD *p≤0.05. Animal numbers: n = 11-17. Statistical test: two-way ANOVA with Bonferroni multiple comparison.

Figure S17. Impact of pharmacological (A-B) and genetic (C-E) modulation of Sigma-1 on exploratory (A, B, D, and E) and locomotor (C) behavior in the open-field test over the first five minutes. A kindling effect on exploratory behavior was observed in E1R and Fenfluramine (FEN) experiments, but pharmacological treatment with E1R or FEN was ineffective in counteracting these effects (A and B). The distance moved in vehicle- and E1R-treated ICR (CD-1) wildtype (WT) and Sigma-1 knockout mice (Sig1R-KO) was unaffected by the kindling process (C). However, the genotype affected exploratory behavior, with Sig1R-KO mice spending more time in the wall zone (D) and less time in the center zone (E). All data are presented as individual data points and mean ± SD *p≤0.05. Animal numbers per group: n = 11–17. Statistical tests: unpaired t-test and one-way ANOVA (A, B) and two-way ANOVA with Bonferroni multiple comparison test (C-E).
