# Supplementary material for: Effects of fenfluramine and sigma‐1‐dependent pharmacological and genetic modulation in a mouse kindling model
Source: Epilepsia. 2026 Apr 29;67(8):4292–304. doi: 10.1002/epi.70270 (PMC13525583; doi:10.1002/epi.70270)
Supplement: Supplementary file 2 [file EPI-67-4292-s002.docx]

**Effects of Fenfluramine and Sigma-1-dependent pharmacological and genetic modulation in a mouse kindling model**

Eva-Lotta von Rüden*^1^, Verena Buchecker*^1^, Amelie Wagner^1^, Victoria M. Stocker^1^, Liga Zvejniece^2^ and Heidrun Potschka^1^

*Both authors contributed equally

***Supplementary Information***

**Supplementary Materials & Methods**

**Supplementary Results**

**Supplementary References**

**Materials and Methods**

## *Animals, housing conditions and breeding*

All experimental animals were housed in the same room in the animal facility under standardized conditions (22 ± 2 °C, 55 ± 10% humidity, and a 12-hour dark-light cycle [lights-on at 05:00 a.m. (CET) and 06:00 a.m. (CEST) and]) with *ad libitum* access to tap water and standardized laboratory food (Ssniff Spezialdiäten GmbH, Soest, Germany). Once a week, animals received a clean home cage (Standard Makrolon type III cage, Ehret, Emmendingen, Germany), equipped with wood chip bedding (SAFE select, J. Rettenmaier & Söhne GmbH & CO. KG, Rosenberg, Germany), nesting material (Cotton nestlet, Zoonlab GmbH, Castrop-Rauxel, Germany), one square mouse house (Zoonlab GmbH, Castrop-Rauxel, Germany), one wooden stick (Labodia, Yens, Switzerland), and one transparent plastic tunnel (Zoonlab GmbH, Castrop-Rauxel, Germany). All animals delivered from Inotiv were allowed to habituate for a minimum of seven days before the start of the experiments. During habituation, mice were accustomed daily to tunnel handling and fixations by the main experimenter and animal caretaker. Before surgery and as long as possible, animals were housed in groups of 3-5 animals per cage. However, upon observation of aggressive or barbering behavior, all animals were separated and housed individually.

As part of a cooperation with Welab, homozygous breeding pairs of Sig1R-KO mice (CD1.129-Sigmar1tm1Lmon/Cnbc) (1), were provided for in-house breeding. Litters were weaned between postnatal day 21 and 28 and subsequently ear-marked for identification and genotyping. The corresponding ICR (CD-1) WT mice were delivered at 4-5 weeks of age to ensure a comparable acclimatization period and were also ear-marked for identification and genotyping, and for blinding purposes.

The commercial breeder provided a health certificate confirming that the animals were free of relevant pathogens, in accordance with FELASA recommendations (Specific-pathogen-free mice, SPF). In-house bred animals were subject to standardized hygiene monitoring conducted at our institute. Based on a standardized score sheet, the general health status was monitored once daily, focusing on general condition, appearance, bodyweight, activity levels, motor function, postoperative wound healing, as well as the occurrence of spontaneous (non-experimentally induced) diseases. Scoring was conducted using a scale from 0 to 3 (0 = physiological state, 3 = humane endpoint) for the described parameters. The humane endpoint is reached when a single parameter scores 3 or when the sum score is ≥5. Once this humane endpoint was reached, affected animals were euthanized.

*Pilot study: Results from coordinate defining*

In a pilot study, stereotaxic coordinates for electrode implantation into the right amygdala were identified and validated for WT and Sig1R-KO mice. Based on these test surgeries, the following coordinates were selected: Female WT and Sig1R-KO (ap: -1,1, lat: +3,2, dv: -5,0) and male WT and Sig1R-KO (ap: -1,5, lat: +3,4, dv: -5,2).

The stereotaxic coordinates for female NMRI mice were selected based on a prior study of Perez-Perez, Monio-Baca (2) (ap: -1,0, lat: +3,2, dv: -5,2). All coordinates are relative to bregma in mm.

## *Surgery, anesthesia, analgesia, and peri- and postoperative care*

Thirty minutes before craniotomy, the animals received meloxicam (5 mg/kg, subcutaneously [s.c.], Metacam^®^, 0.5% injection solution, diluted in *aqua ad injectabilia*, Boehringer Ingelheim, Ingelheim am Rhein, Germany) and Buprenorphine (0.05 mg/kg, s.c., 0.3 mg/ml Buprenovet^®^ Mutidose, Elanco Animal Health, Bad Homburg, Germany diluted in aqua ad injectabilia (WDT, Germany). Mice were anesthetized with isoflurane (Isofluran CP, CP-Pharma, Handelsgesellschaft mbH, Burgdorf, Germany) in 100% oxygen (Linde, Gases Division, Pullach, Germany). Isoflurane concentrations amounted to 4% for induction and 1.8-2.2% for maintenance. The stage of anesthesia was determined by checking the rectal temperature and the toe pinch reflex at regular intervals. To perform the surgical procedure, mice were fixed in a stereotactic device (TSE Systems GmbH, Bad Homburg, Germany) and placed on a heating mat (Dragon Deluxe Heizmatte, 15 x 25 cm and 40 x 60 cm, Reptilienkosmos.de, Niederkrüchten, Germany). Eyes were lubricated with eye ointment (Bepanthen® Augen- und Nasensalbe, Bayer Vital GmbH, Leverkusen, Germany). For local anesthesia, Bupivacaine with epinephrine (8 mg/kg, s.c., Bupivacain 0,5% mit Epinephrin 0,0005% Jenapharm^®^ Injektionslösung; Jenapharm GmbH & Co. KG, Jena, Germany) was applied 15 minutes before skin incision and directly on the exposed skull during surgery. Using a dental drill (Hängebohrmotor, Fischer Goldschmiedebedarf, Pforzheim, Germany), small holes were drilled in the skull to implant a depth stimulation and recording electrode in the right amygdala and to fix the implant with three additional screws (DIN 84 V2A M1,2X2 Schrauben-Preisinger, Munich, Germany). After fixing the bipolar Teflon-isolated stainless-steel electrode and screws with dental acrylic cement (Paladur powder, Kulzer GmbH, Hanau, Germany), skin margins were adapted with sutures (5/0, Surgicryl monofilament, metric 1-5/0, DS12, 45cm, SMI, St.Vith, Belgium). To compensate for fluid loss, a warmed Ringer lactate solution (10 ml/kg, s.c.; B. Braun SE, Melsungen, Germany) was injected at the end of surgery. Following surgery, mice received meloxicam every 12 to 24 hours for three consecutive days.

## *Amygdala kindling*

## *Initial afterdischarge threshold determination*

The initial afterdischarge threshold (ADT) was evaluated after a two-week convalescence period. Therefore, the animals were stimulated with an electrical monophasic square wave pulse (50 Hz for approximately 0.8 sec). Starting at 8 µA, the current was gradually increased by 20% every 60 seconds. The initial ADT is defined as the stimulus intensity in µA that leads to the occurrence of an electrographic discharge pattern in the electroencephalogram (EEG). By definition, the amplitude of the afterdischarge pattern must be twice the amplitude of the EEG at baseline. The afterdischarge pattern is usually accompanied by focal motor seizure activity, or very rarely, it occurs without motor seizure activity. The initial ADT was determined twice, initially without treatment (initial ADT without substance) and a second time after the animals were allocated to treatment groups based on their initial afterdischarge threshold (stratified randomization) with treatment (initial ADT with substance).

## *Kindling*

With the same electrical impulse and a current intensity of 700 µA, animals were stimulated once daily from Monday to Friday. In the first experiment (E1R, NE-100), mice with an initial ADT ≤1000 µA were included. Mice with initial ADTs of 700-1000 µA received daily kindling stimulations of 840 µA, while those with initial ADTs of ≤700 µA received 700 µA. In all the other experiments (pre-study and experiments 2-4), mice with an initial ADT ≥700 µA were excluded from the kindling process to stimulate all animals with the same stimulus.

For all kindling experiments, the following seizure-associated parameters were documented: seizure severity (SS, based on modified Racine scale (3)), motor seizure duration (SD), and electrographic afterdischarge durations 1 and 2 (ADD1 and ADD2). The motor seizure duration is defined as the time in seconds, from the onset of stimulation to the end of a visible behavioral pattern lasting at least five seconds. ADD is defined as the time (≥5s) from the onset of stimulation to the end of the electrographic discharge pattern in the EEG with a frequency of at least 1 Hz and an amplitude twice the baseline. Rarely, the electrographic discharge pattern changes abruptly. In this case, the time from stimulation to the first change in the pattern refers to ADD1, and the total duration of the epileptiform discharge is defined as ADD2. In case no abrupt discharge change is visible, ADD1=ADD2. Please note that the parameter ADD2 is presented as total afterdischarge duration (ADDT) throughout the manuscript and the Supplementary Information.

## *Post-afterdischarge threshold determination*

At the end of the kindling process, the post-afterdischarge threshold (post-ADT) and seizure parameters at threshold stimulation (SS, SD, and ADD1/ADDT) were determined. The post-ADT is defined as the stimulus intensity in µA that leads to the occurrence of an electrographic discharge pattern in the EEG. As before, starting at 8 µA, the current was gradually increased by 20% increments every 60 seconds. The post-kindling ADT was assessed twice: first with the allocated previous treatment (post-kindling ADT with substance), followed by a two-day washout period and a determination of the post-ADT without the substance.

By definition, the amplitude of the afterdischarge pattern must be twice the amplitude of the EEG at baseline. The generation of a hyperexcitable neuronal network in response to the kindling procedure, the afterdischarge pattern is usually accompanied by general motor seizure activity, or rarely, it occurs with focal motor seizure activity.

*Restimulation*

In experiment 1 (E1R and NE-100), a restimulation phase was initiated 13-14 days after the last administration of the compounds to assess any long-lasting effects on ictogenesis.

## *Effects of vehicle and test compounds in fully-kindled ICR (CD-1) wildtype (WT) and Sigma-1 Knockout (Sig1R-KO) mice*

For these experiments, WT and Sig1R-KO from experiment 3 were used. These animals received vehicle treatment during kindling progression. After completing the behavioral analysis of this experiment, animals were further kindled to exhibit at least ten generalized seizures (Racine IV or V, (3)), which defined them as fully kindled. These generalized seizures did not have to occur consecutively, as the kindled state is often less stable in mice compared to rats. Afterwards, the post-kindling ADT was determined repeatedly (Monday, Wednesday, and Friday) following the staircase procedure as described earlier (2). There was at least one day between vehicle and E1R experiments, and at least 96 hours between two consecutive drug experiments. The ADT was considered stable when the repeatedly determined ADT did not differ more than ±20% (= 3-step-range) and mice reliably presented generalized seizure activity. Following a restimulation with 700 µA, WT and Sig1R-KO mice were treated on alternating days with vehicle (saline) or E1R (10, 25, 35, 50 or 75 mg/kg; pretreatment time of 60 minutes) and the ADT, GST and seizure parameters (SS, SD, ADD1, and ADDT) were evaluated following the staircase stimulation procedure with a 20% increase in current intensity. Please note that data were obtained from two different experimental groups, but they were treated in the same way.

## *Behavioral tests*

Behavioral tests were performed in the morning hours, starting from 11:30 a.m. (CET) / 12:30 a.m. (CEST). The saccharin preference test was conducted in the home cage, while the open-field test was assessed and video-documented in a special test room under standardized conditions (temperature 22 ± 2 ̊ C, humidity 40-60 %, lighting 20 lux)

*Saccharin preference test*

Anhedonia-associated behavior was assessed using the saccharin preference test (4) following the kindling procedure. To avoid any side preferences, two water bottles were attached to the cage during the entire study. The observation period comprised four consecutive days (24 h periods) during which the bottles were each filled with 200 g of liquid. On day one, animals were provided with two bottles of tap water to evaluate baseline values. On the following day, one of the two bottles was filled with a 0.1% saccharin solution (Aldrich Saccharin ≥98%, Sigma-Aldrich Chemie GmbH, Taufkirchen, Germany) to assess the preference for sweet solutions. On day three, both bottles were again filled with tap water. During the last day, the bottle on the other side was filled with a 0.1% saccharin solution. Saccharin consumption in % was calculated as the amount of saccharin solution consumed relative to the amount of tap water consumed.

*Open-field test round shape*

The open-field test was conducted to assess locomotor activity and exploratory behavior in an unfamiliar environment (5). At least 30 minutes prior to testing, animals were placed in the test room for habituation. To test two animals in parallel, two black round shaped open-field arenas (diameter: 60 cm; height: 40 cm, lighting: 20 lux) were used simultaneously. The animals were placed individually into the arena, facing the wall at a distance of 10 cm. Between trials, the arenas were cleaned with 0.1% acetic acid solution. 15-minute-long trials were video-recorded using the tracking software EthoVision XT 8.5 (Noldus, Wageningen, the Netherlands; RRID:SCR_000441). The software was used for analyzing locomotor activity as well as the time spent in different areas (wall, center). Particular attention was given to the selected parameters during the first 5 minutes, representing the exploratory phase.

## *Euthanasia and tissue preparation*

At the end of the experiment, animals were euthanized with Pentobarbital (600 mg/kg, i.p., Narkodorm 200mg/ml, diluted in saline, CP Pharma, Burgdorf, Germany). Thirty minutes prior to euthanasia, they received Metamizole (Vetalgin^®^ 500 mg/ml, 100 mg/kg diluted in 0.2% saccharin, per os; MSD Tiergesundheit, Oberschleißheim, Germany) for analgesia. Following euthanasia, the animals were transcardially perfused with 4% paraformaldehyde (PFA) in 0.1 M phosphate buffer (pH 7.4). After post fixation in 4% PFA for a maximum of 24 hours, brains were transferred into 30% sucrose solution for cryoprotection, embedded in Tissue-Tek, and stored at −80 °C until further processing. Five series of coronal sections (40 µm) were cut on a cryostat (HM560M, Microm International GmbH, Walldorf, Germany) and stored at -20°C in a cryoprotectant solution (glycerol and 0.1 M phosphate buffer, pH 7.4, 1/1 in volume). To confirm the correct electrode localization in the right amygdala, one series of the sections was stained with thionine acetate (9937, Carl Roth, Germany).

## *Genotyping*

Based on the protocol from Langa et al. (1) for PCR-based genotyping, two different sets of oligonucleotides were established. The oligonucleotides MS1 (GenBank AF030199, nucleotide positions 2567–2584) and MS3 (GenBank AF030199, nucleotide positions 3726–3708) were used to amplify a 1160‐bp DNA band and adapted to a institute-specific protocol corresponding to the wild‐type mSR1 allele (PCR conditions: Phir Tissue Direct PCR Master Mix (Thermo Fisher), 5 min at 98°C, followed by 32 cycles: 5 s at 98°C, 5 s at 61,5°C and 20 s at 72°C, and, as a final extension step, 1 min at 72°C).

The oligonucleotides S1B (GenBank AF030199, nucleotide positions 3074–3103) and S3A (derived from the lacZ gene in pHM2, GenBank X76683, nucleotide positions 2506–2480) were used to amplify a 529‐bp DNA band and adapted to the protocol corresponding to the mutant mSR1‐null allele (PCR conditions: Phir Tissue Direct PCR Master Mix (Thermo Fisher), 5 min at 98°C, followed by 45 cycles: 5 s at 98°C and 5s at 72°C and 20 s at 72°C, and, as a final extension step, 1 min at 72°C).

## *Statistics*

*Sample size calculation.* For experiments 1, 2, and 4, a sample size of n = 11 per group was calculated based on an effect size of 0.4 (Cohen’s d, main parameter: percentage of sweetened solution consumed out of total fluid intake) using an F test, ANOVA fixed effects, omnibus, one-way (1-β = 0.8 and α = 0.05). However, due to side effects of NE-100 at 45 mg/kg, we decided to terminate the experiment and thus did not achieve the expected sample size for the combination experiments with NE-100 and E1R. For experiment 3, we considered an effect size of 0.42 relevant (Cohen’s d, main parameter: post-kindling ADT) using an RM ANOVA design (1-β = 0.8 and α = 0.05). See Figure S1 for detailed information on exclusion or loss of animals during the course of the experiments.

*Randomization.* Using R software (6) and an in-house simple randomization script, the order for surgery was randomized. This order was maintained for the kindling procedure, injections, and behavioral tests. During vehicle-drug experiments, the order was maintained for the animals that remained for those experiments. Following determination of the initial afterdischarge threshold, a stratified randomization was conducted using an in-house script for stratified randomization.

*Statistical analysis.* Statistical analysis was performed using GraphPad Prism (Version 10, GraphPad, San Diego, CA, USA) and R 4.5.0 for Windows, utilizing the lme4, lmerTest, and emmeans packages. Group differences during kindling acquisition were detected with one-way, two-way, or three-way ANOVA for repeated measurements (dependent factor: time, treatment, genotype) comparing the individual stimulation days for the predefined group comparisons of interest (WT vs. KO; WT Vehicle vs. WT E1R; KO Vehicle vs. KO E1R) using either Bonferroni’s multiple comparisons test or Dunnett’s post-hoc test. The course of kindling was analyzed using a mixed-effect model. Non-parametric data were analyzed using the Kruskal–Wallis test with Dunn’s multiple comparisons. A Fisher’s exact test was used to analyze the proportion of animals that reached generalized seizure stages at the end of the kindling phase. The delta change was calculated by comparing the groups receiving compounds with their respective vehicle control groups. The differences between pre- and post-kindling ADTs and seizure parameters at threshold stimulation with and without prior administration of the test compounds were calculated (delta pre- / post-kindling ADT) and analyzed with either one-way ANOVA with Dunnett’s post-hoc test, unpaired t-test with or without Welch’s correction, or Wilcoxon matched-pairs signed rank test. We first determined if kindling has an impact on behavior in the saccharin preference and open-field test using an unpaired t-test (experiments 1 and 4) or a two-way ANOVA (experiment 3). In case of a significant impact of kindling, the data were further analyzed using ANOVA followed by Bonferroni’s multiple comparisons to evaluate the effects of the test compounds.

*Script for simple randomization*

if (!require(openxlsx)) install.packages('openxlsx')

library(openxlsx)

data <- read.xlsx(file.choose())

number_of_groups <- n

size_of_groups <- c(n)

output <- data.frame(ID=sample(data[,'ID']),group=rep(1:number_of_groups,size_of_groups) )

if (number_of_groups>1) {

output <- output[match(data$ID, output$ID),]

}

output[,3] <- NA ; names(output)[3] <- "Created using the script 'Simple randomisation.R' on:"

output[1,3] <- date()

setwd(choose.dir(default = "", caption = "Select folder"))

write.xlsx(output, file=paste("Simple randomization output ",Sys.Date(), ".xlsx",sep=""), overwrite=F)

*Script for stratified randomization*

if (!require(openxlsx)) install.packages('openxlsx')

library(openxlsx)

data <- read.xlsx(file.choose())

IDS <- data$ID

number_of_groups <- n

size_of_groups <- c(n,n)

temp <- data.frame(groups=rep(1:number_of_groups,size_of_groups), set=rep(1:number_of_groups, length.out=NROW(data)))

data <- data[order(data$variable),]

data <- cbind(data, temp[order(temp$set),])

for(i in 1:length(unique(data$set))){

data[data[,"set"]==i,"groups"] <- sample(data[data[,"set"]==i,"groups"])

}

output <- data[,c(1:3)]

if (number_of_groups>1) {

output <- output[match(IDS, output$ID),]

}

output[,4] <- NA ; names(output)[4] <- "Created using the script 'Stratified randomisation.R' on:"

output[1,4] <- date()

setwd(choose.dir(default = "", caption = "Select folder"))

write.xlsx(output, file=paste("Stratified randomization output ",Sys.Date(), ".xlsx",sep=""), overwrite=F)

*Script for stratified randomization with two variables*

if (!require(openxlsx)) install.packages('openxlsx')

library(openxlsx)

data <- read.xlsx(file.choose())

IDS <- data$ID

number_of_groups <- n

size_of_groups <- c(n,n,n,n)

temp <- data.frame(groups=rep(1:number_of_groups,size_of_groups), set=rep(2:number_of_groups, length.out=NROW(data)))

data <- data[order(data$variable,data$variable2),]

data <- cbind(data, temp[order(temp$set),])

for(i in 2:length(unique(data$set))){

data[data[,"set"]==i,"groups"] <- sample(data[data[,"set"]==i,"groups"])

}

output <- data[,c(1:5)]

if (number_of_groups>1) {

output <- output[match(IDS, output$ID),]

}

output[,5] <- NA ; names(output)[5] <- "Created using the script 'Stratified randomisation.R' on:"

output[1,5] <- date()

setwd(choose.dir(default = "", caption = "Select folder"))

write.xlsx(output, file=paste("Stratified randomization output ",Sys.Date(), ".xlsx",sep=""), overwrite=F)

**Results**

*Impact of Sigma-1 antagonism on effects of E1R*

### *Pre-study: NE-100 at 35 mg/kg + E1R at 50 mg/kg*

In a preliminary study, co-administration with NE-100 at 35 mg/kg and E1R at 50 mg/kg resulted in a significant increase in the initial ADT compared to vehicle-treated mice (p=0.0139; Figure S4A). In contrast, seizure severity, as well as seizure and total afterdischarge duration remained unaffected (Figure S4B, C and D). During kindling acquisition, co-administration of 35 mg/kg NE-100 did not have a relevant impact on effects of E1R on the development of seizure severity. Thus, despite the combination with the Sigma-1 antagonist, E1R significantly delayed the progression of kindling with an impact on seizure severity (time effect p<0.0001, treatment effect p<0.0001; Figure S5A) and on single days with an impact on motor and total electroencephalographic seizure durations (SD: time effect p=0.0031, treatment effect p<0.0001, Figure S5B; ADDT: time effect p=0.018, treatment effect p<0.0001, Figure S5C). These findings are further supported by a significantly lower number of animals presenting generalized seizures (p=0.0007; Figure S5D), a reduced cumulative motor and electroencephalographic seizure duration (p<0.0001 and p<0.0001, respectively; Figure S5E and F). During the post-kindling evaluation, the ADT and seizure parameters at threshold stimulation remained unchanged (Figure S6A-D).

### *Effects of E1R in fully kindled WT and Sig1R-KO mice*

*ADT determination*

Effects of E1R were also determined in fully kindled WT and Sig1R-KO mice (Figure S11). Genetic deficiency had limited effects on the response to E1R. Surprisingly, 10 mg/kg slightly decreased the ADT in Sig1R-KO mice (p_10_=0.0173). Higher doses of E1R (50 and 75 mg/kg) increased the ADT in WT mice. In contrast, in Sig1R-KO mice, a drug effect was only evident with exposure to 75 mg/kg E1R (WT: p_50_=0.005, p_75_=0.0418; Sig1R-KO: p_75_=0.0003; Figure S11A). Seizure severity proved to be reduced by 25, 35, and 75 mg/kg E1R in Sig1R-KO mice and by 75 mg/kg in WT mice (WT p_75_=0.0039; Sig1R-KO: p_25_=0.0313, p_35_=0.0098, p_75_=0.0039; Figure S11B). Motor and electroencephalographic seizure activity remained unaffected regardless of the genotype (Figure S11C and D).

*GST determination*

In cases where the ADT was not associated with generalized tonic-clonic seizure activity, stimulation was gradually increased until a generalized seizure was induced (Figure S12). The genetic deficiency had no relevant impact on the effects of 35, 50, and 75 mg/kg E1R on generalized seizure thresholds (35 mg/kg: p_WT_=0.0304, p_KO_=0.0190; 50 mg/kg: p_WT_=0.0093, p_KO_=0.0108; 75 mg/kg: p_WT_=0.0059, p_KO_=0.0038; Figure S12A). Treatment with 25 mg/kg E1R resulted in a slight decrease in the threshold in WT mice (p=0.0237), but not in Sig1R-KO mice.

At higher doses (50 and 75 mg/kg), E1R increased the duration of motor and electroencephalographic seizure activity regardless of the genotype (SD: WT p_50_=0.0004, p_75_=0.0048; Sig1R-KO p_50_=0.0108, p_75_=0.0237; ADDT: WT p_50_=0.0006, p_75_=0.0092; Sig1R-KO p_75_=0.0389; Figure S9B and C). At lower doses, the impact of E1R and of the genetic deficiency was inconsistent (SD: WT p_10_=0.0342, p_25_=0.0088; ADDT: WT p_25_=0.0079; Sig1R-KO p_35_=0.0198; Figure S12B and C).

Please note that the influence of the genetic deficiency in fully kindled mice was not analyzed for fenfluramine, as we did not observe relevant effects of fenfluramine in the kindling paradigm in our recent study (2).

### *Effects of pharmacological and genetic Sigma-1 modulation on anhedonia-associated behavior, locomotion, and exploration*

Across experiments with pharmacological Sigma-1 targeting, the effects of kindling on saccharin preference were inconsistent, with a kindling-associated reduction in consumption only observed in the experiment focused on E1R (vehicle / E1R / NE-100: p = 0.0057; Figure 6A and B). Regardless of the dose, none of the drugs exerted a relevant effect on this kindling-associated decrease in saccharin consumption (Figure 6A).

In the experiments focused on genetic Sigma-1 modulation, both kindling and genetic deficiency reduced the consumption of saccharin (p_Genotype_=0.0118 and p_Kindling_=0.005, respectively; Figure S15), with post hoc analysis revealing a pronounced effect in vehicle-treated and kindled Sig1R-KO mice (p=0.0021 versus non-kindled sham Sig1R-KO and p=0.0022 versus vehicle-treated and kindled WT mice). Surprisingly, E1R exposure normalized this effect in Sig1R-KO mice (p=0.0324; Figure S15).

The open-field test was conducted over a total duration of 15 minutes to assess locomotor activity and exploratory behavior (Figure S16). All parameters were additionally analyzed during the initial 5-minute interval (Figure 6C-F and S17). Throughout all experiments and analysis intervals, the distance moved was unaffected by the kindling process or by genetic deficiency (Figure 6C and E, Figure S16A, D, G, and Figure S17C). When analyzing exploratory behavior, kindling exhibited minor effects in E1R and fenfluramine experiments within the initial five minutes (E1R p_center_=0.0414 and fenfluramine p_center_=0.0159 and p_wall_=0.0147; Figure S17A and B and Figure 6F). In both experiments, pharmacological treatment was ineffective to counteract these effects.

In Sig1R-KO mice, the Sigma-1 deficiency resulted in significant effects on the exploratory behavior with animals spending more time in the wall zone and less time in the center zone compared to the respective WT group (total 15 minutes: ‘wall’ p_sham/vehicle/E1R_ <0.0001 and center p_sham_=0.0015, p_vehicle_=0.0441 and p_E1R_<0.0001; first five minutes: ‘wall’ p_sham_=0.0007, p_vehicle_=0.00054 and p_E1R_<0.0001 and ‘center’ p_sham_=0.0357 and p_E1R_=0.0115; Figures S16H and I and S17D and E).

# **References**

1.	Langa F, Codony X, Tovar V, Lavado A, Gimenez E, Cozar P, et al. Generation and phenotypic analysis of sigma receptor type I (sigma 1) knockout mice. Eur J Neurosci. 2003;18(8):2188-96.

2.	Perez-Perez D, Monio-Baca C, von Ruden EL, Buchecker V, Wagner A, Schonhoff K, et al. Preclinical efficacy profiles of the sigma-1 modulator E1R and of fenfluramine in two chronic mouse epilepsy models. Epilepsia. 2024;65(8):2470-82.

3.	Racine RJ. Modification of seizure activity by electrical stimulation. II. Motor seizure. Electroencephalogr Clin Neurophysiol. 1972;32(3):281-94.

4.	Klein S, Bankstahl JP, Loscher W, Bankstahl M. Sucrose consumption test reveals pharmacoresistant depression-associated behavior in two mouse models of temporal lobe epilepsy. Exp Neurol. 2015;263:263-71.

5.	Kraeuter AK, Guest PC, Sarnyai Z. The Open Field Test for Measuring Locomotor Activity and Anxiety-Like Behavior. Methods Mol Biol. 2019;1916:99-103.

6.	Team RC. R: A language and environment for statistical computing. R foundation for statistical computing, Vienna, Austria. 2021.
